# Optical Stimulation of Zebrafish Hair Cells Expressing Channelrhodopsin-2

**DOI:** 10.1371/journal.pone.0096641

**Published:** 2014-05-02

**Authors:** Bryan D. Monesson-Olson, Jenna Browning-Kamins, Razina Aziz-Bose, Fabiana Kreines, Josef G. Trapani

**Affiliations:** 1 Department of Biology, Amherst College, Amherst, Massachusetts, United States of America; 2 Neuroscience Program, Amherst College, Amherst, Massachusetts, United States of America; Institut Curie, France

## Abstract

Vertebrate hair cells are responsible for the high fidelity encoding of mechanical stimuli into trains of action potentials (spikes) in afferent neurons. Here, we generated a transgenic zebrafish line expressing Channelrhodopsin-2 (ChR2) under the control of the hair-cell specific *myo6b* promoter, in order to examine the role of the mechanoelectrical transduction (MET) channel in sensory encoding in afferent neurons. We performed *in vivo* recordings from afferent neurons of the zebrafish lateral line while activating hair cells with either mechanical stimuli from a waterjet or optical stimuli from flashes of ∼470-nm light. Comparison of the patterns of encoded spikes during 100-ms stimuli revealed no difference in mean first spike latency between the two modes of activation. However, there was a significant increase in the variability of first spike latency during optical stimulation as well as an increase in the mean number of spikes per stimulus. Next, we compared encoding of spikes during hair-cell stimulation at 10, 20, and 40-Hz. Consistent with the increased variability of first spike latency, we saw a significant decrease in the vector strength of phase-locked spiking during optical stimulation. These *in vivo* results support a physiological role for the MET channel in the high fidelity of first spike latency seen during encoding of mechanical sensory stimuli. Finally, we examined whether remote activation of hair cells via ChR2 activation was sufficient to elicit escape responses in free-swimming larvae. In transgenic larvae, 100-ms flashes of ∼470-nm light resulted in escape responses that occurred concomitantly with field recordings indicating Mauthner cell activity. Altogether, the *myo6b*:ChR2 transgenic line provides a platform to investigate hair-cell function and sensory encoding, hair-cell sensory input to the Mauthner cell, and the ability to remotely evoke behavior in free-swimming zebrafish.

## Introduction

In the auditory, vestibular, and lateral-line systems, the hair cell is the sensory receptor responsible for encoding mechanical stimuli into trains of action potentials in afferent neurons [1], [2]. This mechanotransduction process is accomplished with incredible sensitivity and precision, involving a complex interplay of intrinsic hair-cell mechanisms [3]–[5]. At least two important features of hair cells ensure the speed and precision of sensory transduction, the mechanoelectrical transduction (MET) channel and the ribbon synapse. While much is known about the properties of these two features, how the two contribute to the overall encoding of sensory stimuli is not as well understood.

Located at the tips of hair-cell stereocilia, the MET channel opens within 50 µs following deflection, making it one of the fastest gating channels in the nervous system [3], [6]–[8]. In addition to its rapid gating kinetics, the MET channel has a very large single channel conductance [9]. Combined, these properties of the MET channel allow for graded deflections of stereocilia to result in quick and robust changes in the hair-cell membrane potential. This change in receptor potential rapidly activates voltage-gated calcium channels at the hair-cell ribbon synapse and results in precisely timed fusion of synaptic vesicles and release of glutamate into the synaptic cleft [5].

Due to the rapid and continuous nature of hair-cell stimuli, the ribbon synapse is required to provide the requisite release of glutamate [7]. The ribbon synapse is characterized by the ribbon body, an electron dense structure that is thought to both coordinate synaptic vesicles and allow for their sustained fusion with high temporal fidelity [10]–[13]. Together, the ribbon synapse and the MET channel function to encode sensory information rapidly and faithfully. Here, we sought a means to examine the MET channel's contribution to the encoding of action potentials in afferent neurons. To accomplish this, we turned to optogenetics in order to depolarize the receptor potential without activating MET channels and then compared optically evoked activity with responses to mechanical stimuli.

In order to depolarize hair cells while circumventing activation of the MET channel, we generated a transgenic zebrafish line with hair-cell specific expression of Channelrhodopsin-2 (ChR2) using the *myo6b* promoter [14]. ChR2 is a light-gated ion channel maximally excited by ∼470-nm wavelength light [15], [16]. When expressed in neurons, flashes of ∼470-nm light open ChR2 channels, which then depolarizes the cell membrane and evokes action potentials [15]–[17]. Previously, ChR2 has been expressed in various zebrafish neurons, including those to elicit escape responses and control eye moment [18]–[20]. Here, to examine the contribution of hair-cell mechanisms to the encoding of action potentials in afferent neurons, we performed *in vivo* recordings from afferent neurons of the lateral line in transgenic *myo6b*:ChR2 zebrafish. We compared afferent neuron activity during hair-cell excitation by mechanical activation of MET channels with a waterjet and by optical activation of ChR2, effectively circumventing the MET channel.

In addition to studying the role of the MET channel in afferent spike encoding, we examined behavioral escape responses of transgenic larvae during remote activation of transgenic hair cells. In teleost fish, the fast-start escape response is generated by the Mauthner cells (M-cells), a pair of large reticulospinal neurons in the hindbrain that are excited by sensory inputs including afferent neurons from the ear and lateral line [21]–[25]. Optical stimulation of ChR2-expressing hair cells with flashes of ∼470-nm light generated an escape response that was observed coincidentally with field potentials indicating an M-cell response evoked from free-swimming fish. By shortening the duration of optical stimuli, we found that the escape response and the coincident field potential disappeared when stimuli were less than 100 milliseconds (ms). Altogether, our findings highlight both the vital role of MET channels in the faithful encoding of hair-cell stimuli into action potentials in afferent neurons, and the use of optophysiology to study hair-cell evoked behaviors in zebrafish.

## Materials and Methods

### Ethics statement

All animal protocols were approved by the Institutional Animal Care and Use Committee (IACUC) at Amherst College under assurance number 3925-1 with the Office of Laboratory Animal Welfare. Zebrafish were raised and maintained according to standard procedures [26]. Adults and larvae were maintained under a 14-hour light cycle at 28.5°C.

### Generation of myo6b:ChR2-YFP construct and transgenic line

The *myo6b*:ChR2-YFP plasmid was generated using standard cloning procedures. The *myo6b*-GFP plasmid containing an I-SceI meganuclease cut site was obtained from Dr. Teresa Nicolson at Oregon Health and Science University and the Vollum Institute. The ChR2-YFP plasmid was obtained from Dr. Karl Deisseroth at Stanford University. We identified restriction enzyme sites in the *myo6b* plasmid and removed the GFP sequence via restriction digest. We then ligated the ChR2-YFP sequence into the meganuclease plasmid, resulting in ChR2-YFP expression controlled by the hair-cell specific *myo6b* promoter. The *myo6b*:ChR2-YFP plasmid, along with I-SceI enzyme, was injected into zebrafish embryos at the one cell stage [27]. DNA injections were performed using a pressure injector (MPPI-3, Applied Scientific Instruments, Eugene, Oregon). After DNA injection, F_0_ embryos were identified by visualization of YFP fluorescence, and then raised and outcrossed to wild-type stock. The resulting F_1_ larvae that were screened for hair-cell specific YFP expression were subsequently raised. Adult F_1_ fish were then outcrossed to wild-type fish and ChR2-YFP-positive F_2_ larvae were used for experiments.

### Microscopy and imaging

**Figure 1:**
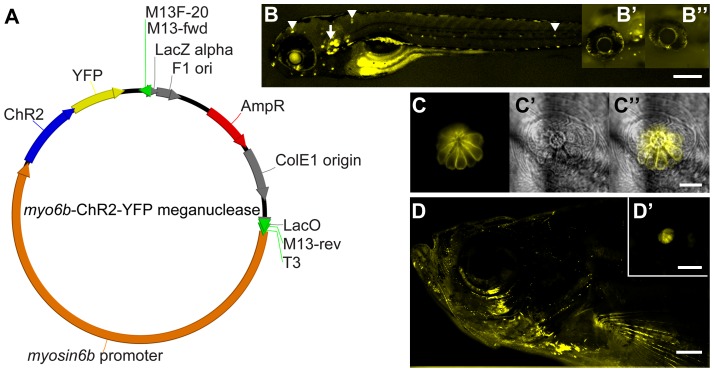
The *myo6b* promoter drives ChR2-YFP expression in hair cells of the ear and lateral line. (**A**) The *myo6b*:ChR2-YFP meganuclease construct was inserted into the zebrafish genome by plasmid injection along with the I-SceI enzyme into the 1-cell stage embryo. (**B**) Hair cells of a 5 days post fertilization (dpf) zebrafish larva express YFP (pseudo-colored yellow) in the lateral line and ear (arrow). The lateral line is composed of neuromasts, clusters of hair cells, at regular intervals around the head and the length of the fish (examples at arrowheads, scale bar 0.05 mm). **B′** ChR2-YFP larval eye at 5 dpf. **B″** Wild-type larval eye at 5 dpf. (**C**) YFP expression in neuromast hair cells. **C′** DIC image of the neuromast **C″** composite of YFP and DIC images (scale bar 0.0125 mm) (**D**) Adult transgenic fish continue to express ChR2-YFP (background removed for clarity, scale bar 2 mm). **D′** YFP expression in an adult neuromast (scale bar 0.05 mm).

Afferent neuron recordings and imaging were performed using 10X and 40X objectives on a fixed-stage upright microscope (BX51WI; Olympus, Center Valley, Pennsylvania) equipped with a FITC filter cube (Chroma Technology, Bellows Falls, Vermont) and a SOLA fluorescent light source (Lumencor Inc., Beaverton, Oregon). Images were captured using a Spot Pursuit Camera (1.4 MP Monochrome w/o IR; Diagnostic Instruments, Sterling Heights, Michigan) and either the upright or a dissecting microscope (Nikon SMZ 1500; Melville, New York). YFP-fluorescent images were pseudocolored yellow using ImageJ (NIH, Bethesda Maryland) and were adjusted for brightness and contrast using Photoshop CS5 (Adobe, San Jose, California). For the adult transgenic image in Figure 1D, the background surrounding the fish body was removed for clarity using Photoshop CS5. Escape responses were recorded with a Chromachip II Camera, (Javelin Electronics, High Wycombe, United Kingdom) mounted on a dissection microscope (Olympus SZ40; Center Valley, Pennsylvania). To filter the bright flash of blue excitation light from the video, incoming light to the camera was passed through an orange acrylic filter (Pearl Biotech, San Francisco, California). Video output from the camera was collected on a PC computer using a USB-capture device and software (Elgato Video, San Francisco, California).

### Lateral-line electrophysiology

Our recording procedures were previously described in detail [28]. Briefly, larvae were anesthetized, mounted, and microinjected in the heart with 125 µM α-bungarotoxin to block muscle activity (Abcam, Cambridge, Massachusetts). Larvae were then rinsed and returned to normal extracellular solution (in mM: 130 NaCl, 2 KCl, 2 CaCl_2_, 1 MgCl_2_ and 10 HEPES, pH 7.8, 290 mOsm). Extracellular recordings were performed at room temperature with borosilicate glass recording electrodes (Sutter Instruments, Novato, CA) fabricated with long tapers and resistances between 5 and 15 MΩ in extracellular solution (P-97 Puller; Sutter Instruments, Novato, CA). Extracellular action currents were recorded from an individual lateral line afferent neuron in the loose-patch configuration (seal resistances ranged from 20 to 80 MΩ). Recordings were done in voltage-clamp mode, sampled at 50 µs/pt, and filtered at 1 kHz with an EPC 10 amplifier and Patchmaster software (Heka Electronic, Bellmore, New York).

### Mechanical stimulation

Stimulation of neuromast hair cells was performed as previously described [28]. Briefly, mechanical stimuli were delivered to hair cells using a pressure clamp (HSPC-1; ALA Scientific, New York) attached to a glass micropipette (tip diameter ∼30 µm) filled with normal extracellular solution. This waterjet was positioned approximately 100 µm from a given neuromast and the displacement of the neuromast kinocilia was verified by eye. The waterjet pressure clamp was driven by a step voltage command delivered by the recording amplifier via the Patchmaster software. The stimulus pressure was monitored and recorded via a feedback sensor located on the pressure clamp headstage. After establishing a recording from a given afferent neuron, its primary innervated neuromast was identified by progressively stimulating from neuromast to neuromast until phase-locked spiking was observed.

### Optical stimulation

Hair cells of wild type and transgenic zebrafish larvae were optically stimulated using flashes of light from a fluorescent light source (SOLA Light Driver; Lumencor, Beaverton Oregon). White light flashes were subsequently filtered via a narrow-pass FITC excitation filter (460 to 490 nm; Chroma Technology, Bellows Falls, Vermont) and transmitted through a 40X water immersion lens (Olympus, Center Valley, Pennsylvania) onto the mounted larva. Optical flashes were triggered via a 5-volt TTL output from the EPC10 amplifier and Patchmaster software (HEKA Electronik, Bellmore, New York) to a remote control accessory (RCA; Lumencor, Beaverton Oregon) on the SOLA light source. Light intensity at the level of the sample was measured at 6.9-klux using a Light Meter Probe (MLT331; AD Instruments, Colorado Springs, Colorado). For behavioral experiments, light flashes were delivered using a blue LED light (470 nm; LEDSupply.com, Randolph, Vermont) with a Tight Spot LED Optic lens (Carclo, Latrobe, Pennsylvania) connected to a 1,000 mA BuckPuck driver (LEDSupply.com, Randolph, Vermont). Flashes were triggered with a TTL signal from the Powerlab 26T amplifier (AD Instruments, Colorado Springs, Colorado) used for the hindbrain recordings. Light intensity for this behavioral preparation was measured at 3.1-klux.

### Field Recordings

M-cell field recording procedures were based on those from previously published studies [29], [30]. All recordings were performed with larvae in a drop of distilled water at room temperature. For touch stimulation, larvae were stimulated with a waterjet while either embedded in 2% low-melt agarose or free swimming, and we did not observe a significant difference in field potentials between the two (data not shown). For optical stimulation, all larvae were free swimming. Field potentials were recorded with stainless steel insect pins embedded in the bottom of a Sylgard-coated dish (Dow-Corning, Midland, Michigan). Potentials were recorded using an extracellular amplifier (Model 3000; A–M systems Inc, Carlsborg, Washington) with 5000X gain, a 300-Hz high pass filter, and 1-kHz low pass filter and were then collected with a PowerLab 26T (AD instruments, Colorado Springs, Colorado).

### Signal analysis

Data were analyzed using custom software written in Igor Pro (Wavemetrics, Lake Oswego, Oregon) and were plotted with Igor Pro and Prism 5 (Graphpad, La Jolla, California). Data sets were tested for normality using Kolmogorov-Smirnov normality tests. Statistical significance between conditions was determined using paired two-tailed Student's *t* tests. In order to evaluate the fidelity of phase locking for evoked spiking, we converted individual spike times, with time = 0 coinciding to stimulus onset, to unit vectors with appropriate phase angle for the given stimulus and then calculated vector strength (*r*) [31].

## Results

In order to examine the contribution of the MET channel to the rapid and precise encoding of action potentials in afferent neurons, we sought a means to depolarize hair cells without activating MET channels. Therefore, we generated a stable transgenic line of zebrafish expressing the light-activated ion channel, Channelrhodopsin-2 (ChR2), in hair cells.

### Hair cell ChR2 expression in the myo6b:ChR2-YFP transgenic zebrafish

To express ChR2 in hair cells, we generated transgenic zebrafish expressing ChR2 tagged with yellow fluorescent protein (YFP) driven by the *myo6b* promoter (*myo6b*:ChR2-YFP; Fig. 1A). In order to confirm hair-cell expression of ChR2 in larval zebrafish, we analyzed YFP fluorescence from six stable transgenic lines at 5 days post fertilization. For our experiments, we selected the line with the strongest fluorescence in all hair cells of the ear and lateral line (Fig. 1B and 1C). Importantly, we did not observe any obvious misexpression of YFP including any in the larval eye (Fig. 1B′ and 1B″). In addition, YFP fluorescent hair cells were observed as soon as they are deposited along the lateral line (data not shown). Finally, the transgenic line continued to express ChR2-YFP in hair cells during development and throughout adulthood (Fig. 1D). This stable expression provides an exciting opportunity for optogenetic experiments with adult animals. Importantly, we have not observed any changes in adult zebrafish mating, fecundity, or life span.

### Afferent spike encoding following mechanical and optical activation of hair cells

**Figure 2:**
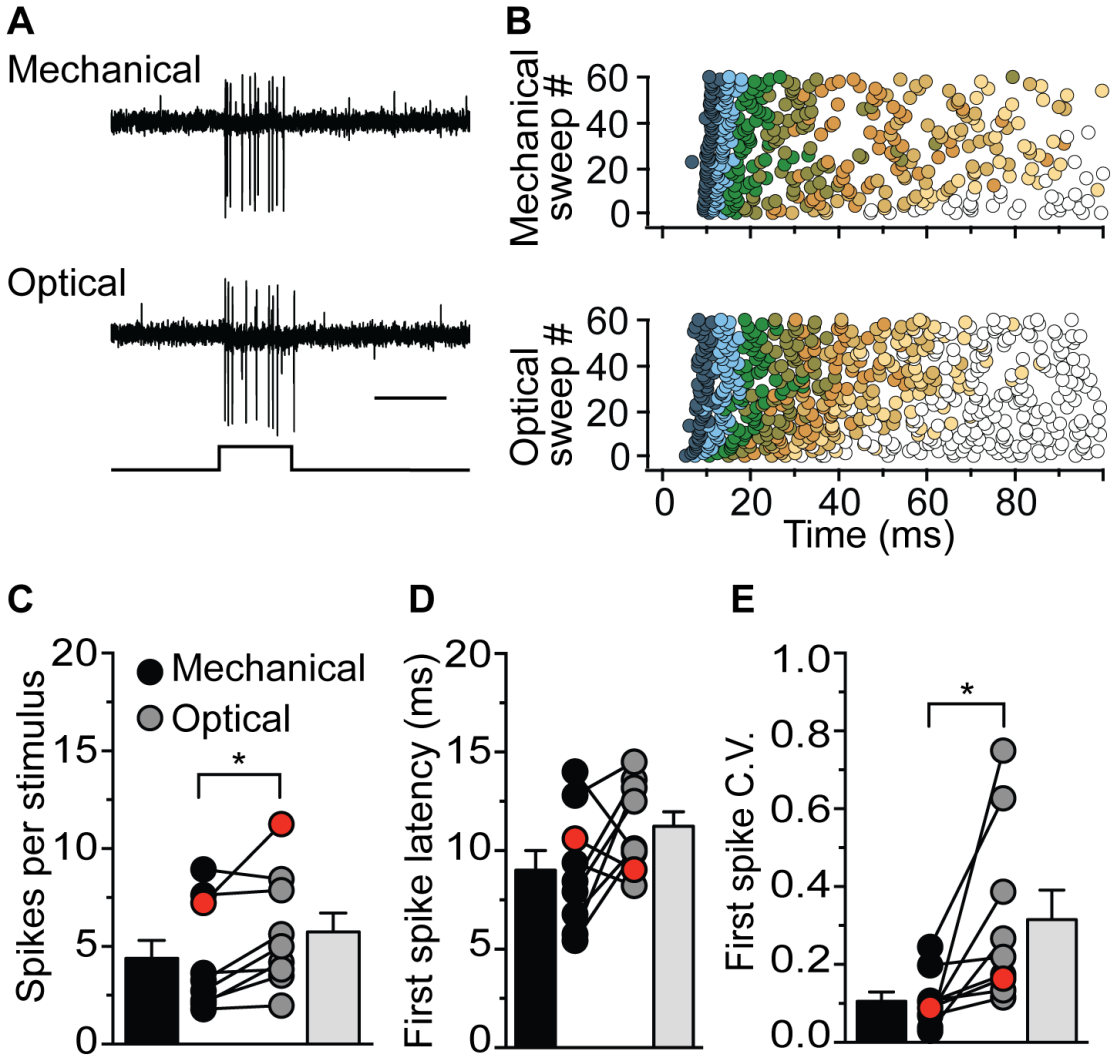
Comparison of spike encoding following mechanical and optical activation of hair cells. (**A**) Mechanical stimulation of neuromast hair cells with a waterjet and optical stimulation with ∼470-nm light produced similar patterns of spiking in the afferent neuron. Stimulus protocol is below the traces (scale bar 100 ms). (**B**) Plots of all spikes in response to 60 repeated sweeps for the cell recorded in A, using either a mechanical (upper) or optical (lower) 100-ms stimulus. Each successive spike is represented by circles of incrementing color: first spike of each sweep is dark blue, the second light blue, the third green, the fourth brown, with subsequent spikes fading from brown to white. (**C**) The mean number of spikes per sweep for cells (n = 9) recorded with both mechanical (black) and optical (grey) stimuli. The overall mean plus SEM across all recorded cells are represented by the bars. The cell labeled red in C, D, and E corresponds to the cell shown in A and B. (**D**) The mean first spike latency for all cells recorded with either mechanical or optical stimuli. Symbols and bars are as in C. (**E**) The coefficient of variation (C.V.) of the first spike latency for all cells recorded with both mechanical and optical stimuli. Symbols and bars are as in C and D.

Using our newly created transgenic line, we performed *in vivo* electrophysiological recordings from single afferent neurons of the lateral line while alternately stimulating transgenic hair cells with either a mechanical waterjet or optical flashes of ∼470-nm light. Both mechanical and optical stimulation using 100-ms square-wave pulses produced similar trains of action potentials (spiking) in afferent neurons (Fig. 2A). Recordings from wild-type larvae showed no responses during presentation of similar flashes of light (data not shown). In order to characterize spike encoding during repeated pulses, we recorded 60 consecutive sweeps during both mechanical and optical stimulation (Fig. 2B). Interestingly, there was a significant increase in the number of spikes per stimulus with optical stimulation (Fig. 2C; mechanical spikes per sweep = 4.4±0.9, optical spikes sweep = 5.7±1.0; n = 9; *p*<0.05).

Auditory features are encoded in afferent neuron spiking using multiple parameters, including total spike number and first spike latency [32]. Total spike number conveys information about the amplitude, frequency, and spatial location of a sound [33], [34]. However, it has been reported that the nervous system must average the total number of spikes across multiple stimulus presentations in order to encode these features accurately [32], [34]. First spike latency, which is the time from stimulus onset to when the first action potential is observed, is also known to encode the amplitude, frequency, and spatial location of stimuli [35], [36]. In addition, first spike latency is not thought to require averaging across repeated stimuli in order to encode these features accurately [32], [34], [36]. Thus, animals may use first spike latency to respond quickly to auditory stimuli [37]. Presumably, in order for this fast encoding to be meaningful, these initial spikes should occur with minimum variability from stimulus to stimulus.

To determine the fidelity of first spike latencies across repeated stimuli, we analyzed recordings of 60 consecutive sweeps during mechanical and optical stimulation. Our analysis revealed no significant difference between mean first spike latency (FSL) during mechanical versus optical stimulation (Fig. 2D; mechanical FSL = 9.0±0.9 ms; optical FSL = 11.2±0.7 ms; n = 9; *p* = 0.11). This result was somewhat surprising, as ChR2 gating has been shown to be significantly slower than MET channel gating [3], [38], though the overexpression of ChR2 may confound our results. Further analysis revealed that the coefficient of variation (C.V.) of first spike latencies was significantly greater with optical stimuli (Fig. 2E; *p*<0.05). One possibility for this result is that depolarization via ChR2 activation is not as consistent as depolarization through collective, and concerted activation of MET channels [39], [40].

### Comparison of phase-locked spiking during mechanical and optical stimulation of hair cells

**Figure 3:**
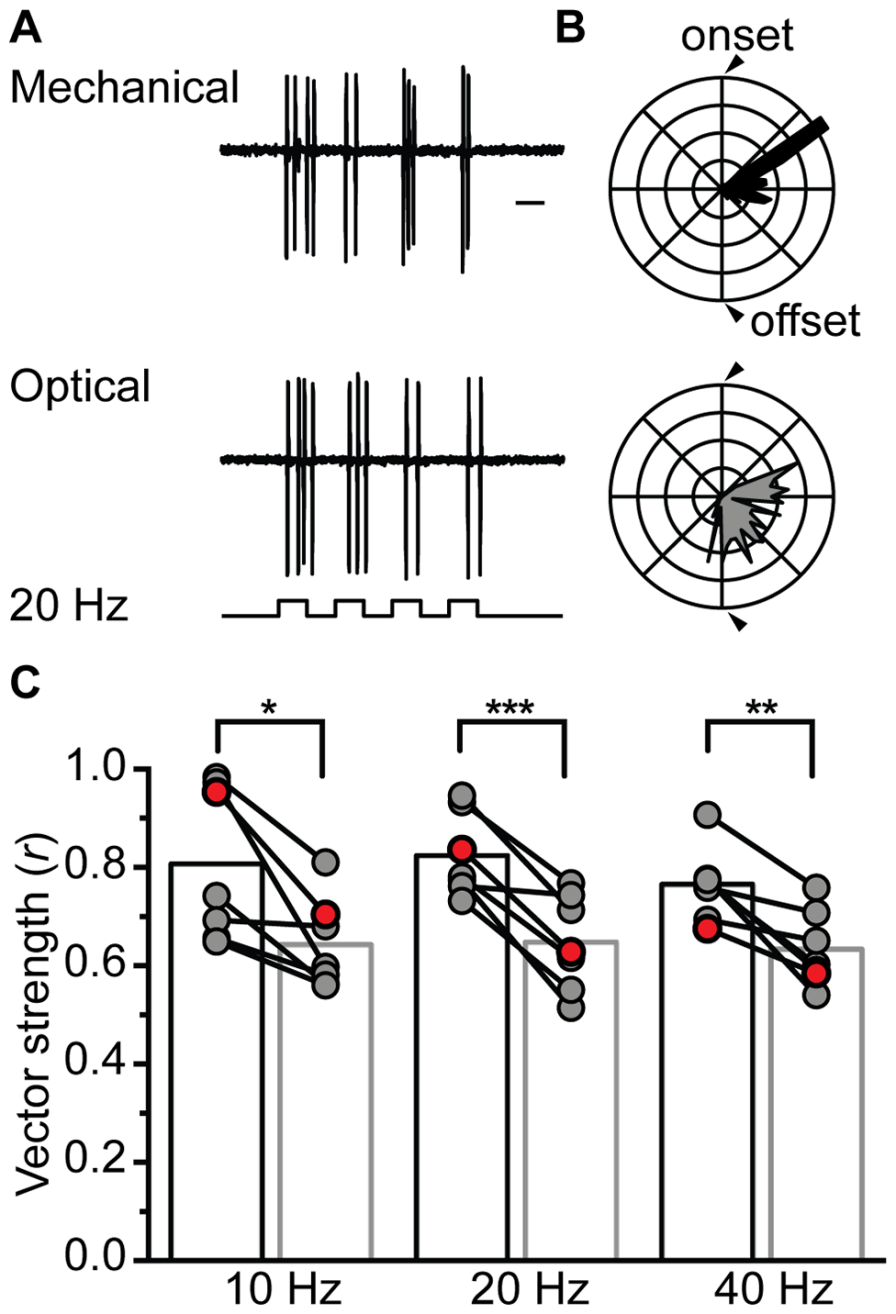
Mechanical stimulation is required for the temporal fidelity of phase-locked spiking. (**A**) *Top*: Phase-locked spiking for a mechanical 20-Hz stimulus. *Middle*: Phase-locked spiking for an optical 20-Hz stimulus. *Bottom*: 20-Hz stimulus protocol (scale bar 25 ms). Note that stimuli were 25-ms in length and delivered at a 20-Hz rate. (**B**) Polar plots from 60 sweeps of mechanical (upper) and optical (lower) stimulation. Plots constructed from all spikes elicited by 20-Hz stimulation of the cell shown in A. (**C**) The vector strength of phase-locked spiking for multiple cells recorded during 60 sweeps of 10, 20, and 40 Hz mechanical and optical stimulation. The bars represent the mean of the vector strength from all cells.

Lateral-line hair cells are capable of encoding higher frequency mechanical stimuli with great accuracy (see [41]). Given the increased variability of first spike latency seen with optical stimuli, we examined the temporal fidelity of spike encoding during 20-Hz mechanical and optical stimulation. Visual inspection of single sweeps revealed that spiking was more closely phase locked to the stimulus onset for mechanical stimuli than for optical stimuli (Fig. 3A). To examine the relationship between the time of stimulus onset and the subsequently evoked spikes, we constructed polar plots with spikes from 60 consecutive sweeps at 20 Hz. Figure 3B shows that the majority of spikes occurred near stimulus onset with mechanical stimulation, whereas when stimulated optically, spiking occurred throughout the duration of the stimulus with some spikes occurring after stimulus offset (Fig. 3B). This experiment provides further evidence that depolarization of hair cells through ChR2 activation is less accurate than the rapid and concerted activation of MET channels with mechanical stimuli.

A reduction in phase locking of spikes in response to delivered stimuli is often quantified by calculating vector strength, *r* [31]. Here, we quantified vector strength during delivery of 10, 20, and 40-Hz mechanical and optical stimuli (Fig. 3C). Across all frequencies, optical activation of hair cells (10 Hz *r* = 0.64±0.04; 20 Hz *r* = 0.65±0.04; 40 Hz *r* = 0.63±0.03; n = 7) led to significantly less accurate encoding than mechanical stimulation (10 Hz *r* = 0.80±0.06, *p*<0.05; 20 Hz *r* = 0.82±0.03, *p*<0.001; 40 Hz *r* = 0.77±0.03, *p*<0.01). Altogether, these results highlight the importance of the MET channel for the faithful encoding of relevant sensory stimuli into action potentials in afferent neurons of the lateral line.

### Hair-cell activation of the escape response

**Figure 4:**
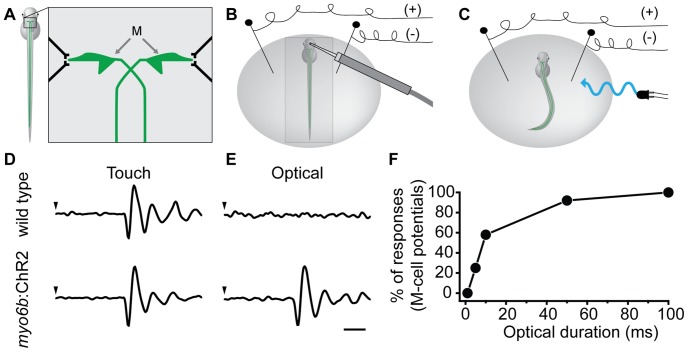
Escape responses from touch and optical stimulation of wild type and *myo6b*:ChR2 transgenic larvae. (**A**) Diagram depicting the Mauthner cells (M), a pair of neurons in the hindbrain of teleost fish. The axons of the M-cells project into the spinal cord where they synapse on primary motor neurons and elements of the central pattern generator responsible for left-right tail motions. (**B, C**) Diagrams of the setup for field recordings of M-cell potentials from larval zebrafish. (**B**) A waterjet was used to stimulate touch receptors on the head of a larva embedded in low melt agarose. (**C**) For optical stimuli, field potentials were collected from free-swimming transgenic larvae. (**D**) In both wild type and *myo6b*:ChR2 transgenic larvae, the M-cell was activated in response to a 100-ms touch stimulus (onset at arrowhead). (**E**) In wild-type larvae, the M-cell was not activated by flashes of ∼470-nm light (n = 18). Transgenic *myo6b*:ChR2 larva responded to ∼470-nm light with both a field potential (n = 55; scale bar 2 ms for D and E) and an escape response (not shown). (**F**) Increasing the duration of optical flashes increased the percentage of observed field potentials (seen in E) and escape responses in transgenic larvae (n = 12). Note that flashes that were 100-ms or greater resulted in 100% success rate for observed escape responses and field potentials.

Hair-cell sensory information is vital to the startle and escape responses in vertebrates. We determined whether remote activation of hair cells with optical stimuli could evoke an escape response in transgenic larvae. Delivery of both touch stimuli with a waterjet and optical stimuli with flashes of ∼470-nm light evoked similar escape responses. In addition, we recorded field potentials in order to determine whether a pattern generated by M-cell activity was similar during escape responses from the two modes of stimulation (Fig. 4B–C). Waveforms from field potential recordings indicated initial M-cell responses, as well as activity from other hindbrain neurons and subsequent contraction of axial muscles [29], [30]. Both wild type and *myo6b*:ChR2 transgenic larvae displayed similar field potentials in response to touch stimuli delivered via a waterjet directed at the head (Fig. 4D). However, wild-type larvae did not respond to a 100-ms flash of ∼470-nm light (Fig. 4E; Video S1; n = 18), while *myo6b*:ChR2 larvae displayed a robust escape response along with concomitant field potentials (Fig. 4E; Video S2; n = 55). If hair-cell inputs were bringing the M-cell membrane potential to threshold, we predicted that shortening the duration of the optical flash would decrease spiking of afferent neurons and thus lower the probability of an M-cell action potential. Consistent with our prediction, we found that by shortening optical stimulus duration, we reduced the frequency of observed escape responses and coincident field potentials (Fig. 4F).

## Discussion

Here, we present an initial characterization of transgenic zebrafish with hair-cell specific ChR2 expression. We successfully evoked stimulus-dependent spiking in afferent neurons with optical activation of transgenic hair-cells using flashes of ∼470-nm light. Additionally, we evoked escape responses with optical stimulation of transgenic hair-cells in free-swimming zebrafish larvae. ChR2 expression in hair cells of the ear and lateral line provide an opportunity for investigation of hair-cell sensory encoding and hair cell stimulus-induced activation of the escape response.

### First spike latencies are more variable during optical stimulation

Utilizing our *myo6b*:ChR2 transgenic line, we found that flashes of ∼470-nm light evoked trains of spiking in afferent neurons that were remarkably similar to the spiking evoked by mechanical stimuli. Unsurprisingly, the total number of spikes in response to each mode of stimulation was significantly different. One logical explanation for the increase in spikes during optical activation is that the overexpression of ChR2 throughout the hair-cell membrane, combined with a high intensity flash of light resulted in an increased amount of depolarization compared to the mechanical stimulus. The greater depolarization of the hair-cell receptor potential would result in increased neurotransmitter release and subsequent spikes in the afferent neuron. Interestingly, despite the increased number of spikes, mechanical stimulation still resulted in similar first spike latencies.

First spike latency is important for encoding the onset of sound [32], [34], [36]. We observed that the mean first spike latency in response to either repeated mechanical or optical stimuli was not different. However, the variability of the first spike latencies during optical stimulation was significantly greater. For mechanical stimulation, a fast, temporally precise change in receptor potential is provided in part by the large conductance of the MET channels, and their localization to the tips of stereocilia, which ensures their simultaneous activation during deflection of the hair bundle [39], [40], [42], [43]. In contrast to this localized, large depolarizing current source, we observed ChR2 channels, via YFP fluorescence, along the entire plasma membrane of the hair cell. Thus, a flash of ∼470-nm light most likely results in stochastic activation of ChR2 channels along the entire cell membrane. This widespread, random activation of a large number of relatively small conductance ion channels could explain the increased variability of first spike latencies.

The increased variability during optical stimulation results from an increase in both faster and slower first spike latencies, which may explain the lack of difference between the mean first spike latency for the two modes of stimulation. The increase in temporal jitter of first spikes during ChR2 activation further highlights the importance of MET-channel activation for the temporal fidelity of spike encoding [3], [6]–[8]. We also observed a difference in the rate of spike adaptation between the two modes of stimulation (see Fig. 2B). ChR2 channels are known to adapt during prolonged stimulation [44]. In addition, there are many sources of adaptation described within the hair cell, including mechanisms involving MET channels [8], [45]. We are currently investigating these mechanisms of hair-cell adaptation.

### The temporal fidelity of phase-locked spiking is reduced during optical stimulation

Given the increased variability of first spike latencies that we observed, we predicted that this would result in decreased vector strength during sustained stimulation. Therefore, we delivered optical stimuli at 10, 20, and 40 Hz, which did result in a significant reduction in the fidelity of phase-locked spiking. Presumably, this occurred for many of the same reasons as for why the first spike latencies were more variable with ChR2 activation. We note that newly optimized versions of ChR2 may be capable of higher fidelity phase locking [46]. For instance, ChETA is capable of following stimuli up to 200 Hz [44]. Altogether, our findings provide evidence that the direct mechanical gating of MET channels plays a role in the faithful encoding of sensory stimuli.

### Escape responses and M-cell activity are evoked by optical activation of hair cells

In teleost fish, the M-cell receives sensory information from the eighth cranial nerve as well as from the lateral line [24], [25], [47], [48]. Here, we show that optical stimulation of hair cells with flashes of ∼470-nm light evoked an escape response in transgenic larvae. Furthermore, reducing the duration of optical stimuli reduced the number of observed escape responses. We also found that recorded field potentials consistent with M-cell activity were lost concomitantly with the loss of the escape response. This result provides strong support that hair cell stimuli evoke escape responses through activation of the M-cell. Our experiments were based on activation of both ear and lateral-line hair cells. While afferent activity from zebrafish auditory hair cells is known to evoke M-cell responses [22], [23], [49], a role in the larval zebrafish is less well described [50]. The precise activation of lateral-line hair cells using the *myo6b*:ChR2 transgenic line will allow for examination of the lateral line contribution to the escape response. Furthermore, the ability to control hair-cell activity remotely in free-swimming larvae provides many opportunities for physiological and behavioral studies in zebrafish.

### Conclusion

Here, we demonstrate the potential for using optogenetics in the study of sensory hair cells and their role in eliciting an escape response. We described transgenic zebrafish with hair-cell specific expression of ChR2. We drove hair-cell activity remotely and recorded encoded spikes from afferent neurons of the lateral line. We compared phase-locked spiking between remote optical and direct mechanical stimulation. We found that temporal precision of phase locking, which is essential to sensory encoding, was lost when the receptor potential was depolarized through ChR2 activation instead of through MET channels. Our findings provide *in vivo* support for the role of the MET channel in the temporally precise encoding of sensory stimuli into spikes in afferent neurons. We also demonstrate how remote activation of ChR2 in hair cells can elicit behavioral escape responses in free-swimming fish. The optical transparency of the zebrafish larva, combined with the power of optophysiology, provide a robust and reliable way to observe and activate hair cells in both the ear and lateral line. Altogether, hair-cell specific ChR2 expression will be a useful tool for research on hair cells and neuronal circuits that involve encoded information from hair cells.

## Supporting Information

Video S1 **Wild-type larval responses to 100-ms flashes of ∼470-nm light.** Three flashes of ∼470-nm light (100 ms each) are delivered to a wild-type larva (5 dpf). Field recordings of similar experiments revealed no Mauthner cell activation during the stimulation. A blue circle is shown in the upper left-hand corner of the video indicating the duration of each flash of ∼470-nm light.
(MP4)

Video S2 **Transgenic** ***myo6b*** **:ChR2 larval responses to 100-ms flashes of ∼470-nm light.** A single *myo6b*:ChR2 transgenic larva (5 dpf) is exposed to three flashes of ∼470-nm light (100 ms per flash). Note that the transgenic larva displays a startle response and ballistic escape to each flash of light. Field recordings of similar experiments indicated Mauthner cell activity with escape responses elicited by ∼470-nm flashes. A blue circle is shown in the upper left-hand corner of the video indicating the duration of each flash of ∼470-nm light.
(MP4)

## References

[1] Hudspeth AJ (1985) The Cellular Basis of Hearing: The Biophysics of Hair Cells. Science 230: 745–752. 2414845 10.1126/science.2414845

[2] Stommel EW, Stephens RE, Alkon DL (1980) Motile statocyst cilia transmit rather than directly transduce mechanical stimuli. J Cell Biol 87: 652–662. 7462319 10.1083/jcb.87.3.652 PMC2110788

[3] Fettiplace R (2009) Defining features of the hair cell mechanoelectrical transducer channel. Pflugers Arch 458: 1115–1123 10.1007/s00424-009-0683-x 19475417 PMC2745616

[4] Nicolson T (2005) The genetics of hearing and balance in zebrafish. Annu Rev Genet 39: 9–22. 16285850 10.1146/annurev.genet.39.073003.105049

[5] Safieddine S, El-Amraoui A, Petit C (2012) The Auditory Hair Cell Ribbon Synapse: From Assembly to Function. Annu Rev Neurosci 35: 509–528 10.1146/annurev-neuro-061010-113705 22715884

[6] Beurg M, Fettiplace R, Nam J-H, Ricci AJ (2009) Localization of inner hair cell mechanotransducer channels using high-speed calcium imaging. Nat Neurosci 12: 553–558 10.1038/nn.2295 19330002 PMC2712647

[7] Fuchs PA (2005) Time and intensity coding at the hair cell's ribbon synapse. J Physiol 566: 7–12 10.1113/jphysiol.2004.082214 15845587 PMC1464726

[8] Ricci AJ, Kennedy HJ, Crawford AC, Fettiplace R (2005) The Transduction Channel Filter in Auditory Hair Cells. J Neurosci 25: 7831–7839 10.1523/JNEUROSCI.1127-05.2005 16120785 PMC6725256

[9] Ricci AJ, Crawford AC, Fettiplace R (2003) Tonotopic variation in the conductance of the hair cell mechanotransducer channel. Neuron 40: 983–990. 14659096 10.1016/s0896-6273(03)00721-9

[10] Goutman JD, Glowatzki E (2007) Time course and calcium dependence of transmitter release at a single ribbon synapse. Proc Natl Acad Sci U S A 104: 16341–16346 10.1073/pnas.0705756104 17911259 PMC2042208

[11] Moser T, Beutner D (2000) Kinetics of exocytosis and endocytosis at the cochlear inner hair cell afferent synapse of the mouse. Proc Natl Acad Sci U S A 97: 883–888. 10639174 10.1073/pnas.97.2.883 PMC15425

[12] Schnee ME, Santos-Sacchi J, Castellano-Muñoz M, Kong J-H, Ricci AJ (2011) Calcium-dependent synaptic vesicle trafficking underlies indefatigable release at the hair cell afferent fiber synapse. Neuron 70: 326–338 10.1016/j.neuron.2011.01.031 21521617 PMC3254016

[13] Zampini V, Johnson SL, Franz C, Lawrence ND, Münkner S, et al (2010) Elementary properties of CaV1.3 Ca(2+) channels expressed in mouse cochlear inner hair cells. J Physiol 588: 187–199 10.1113/jphysiol.2009.181917 19917569 PMC2817446

[14] Obholzer N, Wolfson S, Trapani JG, Mo W, Nechiporuk A, et al (2008) Vesicular glutamate transporter 3 is required for synaptic transmission in zebrafish hair cells. J Neurosci 28: 2110–2118 10.1523/JNEUROSCI.5230-07.2008 18305245 PMC6671858

[15] Nagel G, Ollig D, Fuhrmann M, Kateriya S, Musti AM, et al (2002) Channelrhodopsin-1: A Light-Gated Proton Channel in Green Algae. Science 296: 2395–2398 10.1126/science.1072068 12089443

[16] Nagel G, Szellas T, Huhn W, Kateriya S, Adeishvili N, et al (2003) Channelrhodopsin-2, a directly light-gated cation-selective membrane channel. Proc Natl Acad Sci 100: 13940–13945. 14615590 10.1073/pnas.1936192100 PMC283525

[17] Boyden ES, Deisseroth K, Wang L-P, Zhang F (2006) Channelrhodopsin-2 and optical control of excitable cells. Nat Methods 3: 785+. 10.1038/nmeth936 16990810

[18] Douglass AD, Kraves S, Deisseroth K, Schier AF, Engert F (2008) Escape behavior elicited by single, channelrhodopsin-2-evoked spikes in zebrafish somatosensory neurons. Curr Biol CB 18: 1133–1137 10.1016/j.cub.2008.06.077 18682213 PMC2891506

[19] Schoonheim PJ, Arrenberg AB, Bene FD, Baier H (2010) Optogenetic Localization and Genetic Perturbation of Saccade-Generating Neurons in Zebrafish. J Neurosci 30: 7111–7120 10.1523/JNEUROSCI.5193-09.2010 20484654 PMC3842466

[20] Umeda K, Shoji W, Sakai S, Muto A, Kawakami K, et al (2013) Targeted expression of a chimeric channelrhodopsin in zebrafish under regulation of Gal4-UAS system. Neurosci Res 75: 69–75 10.1016/j.neures.2012.08.010 23044184

[21] Eaton RC, Farley RD, Kimmel CB, Schabtach E (1977) Functional development in the Mauthner cell system of embryos and larvae of the zebra fish. J Neurobiol 8: 151–172 10.1002/neu.480080207 856948

[22] Eaton RC, Lee RKK, Foreman MB (2001) The Mauthner cell and other identified neurons of the brainstem escape network of fish. Prog Neurobiol 63: 467–485 10.1016/S0301-0082(00)00047-2 11163687

[23] Liu KS, Fetcho JR (1999) Laser Ablations Reveal Functional Relationships of Segmental Hindbrain Neurons in Zebrafish. Neuron 23: 325–335 10.1016/S0896-6273(00)80783-7 10399938

[24] Pujol-Martí J, Baudoin J-P, Faucherre A, Kawakami K, López-Schier H (2010) Progressive neurogenesis defines lateralis somatotopy. Dev Dyn 239: 1919–1930 10.1002/dvdy.22320 20549716

[25] Korn H, Faber DS (1975) Inputs from the posterior lateral line nerves upon the goldfish Mauthner cell. I. Properties and synaptic localization of the excitatory component. Brain Res 96: 342–348. 169962 10.1016/0006-8993(75)90745-3

[26] Kimmel CB, Ballard WW, Kimmel SR, Ullmann B, Schilling TF (1995) Stages of embryonic development of the zebrafish. Dev Dyn 203: 253–310 10.1002/aja.1002030302 8589427

[27] Soroldoni D, Hogan BM, Oates AC (2009) Simple and efficient transgenesis with meganuclease constructs in zebrafish. Methods Mol Biol 546: 117–130 10.1007/978-1-60327-977-28 19378101

[28] Trapani JG, Nicolson T (2010) Physiological recordings from zebrafish lateral-line hair cells and afferent neurons. Methods Cell Biol 100: 219–231 10.1016/B978-0-12-384892-5.00008-6 21111219

[29] Issa FA, O'Brien G, Kettunen P, Sagasti A, Glanzman DL, et al (2011) Neural circuit activity in freely behaving zebrafish (Danio rerio). J Exp Biol 214: 1028–1038 10.1242/jeb.048876 21346131 PMC3044078

[30] Prugh JI, Kimmel CB, Metcalfe WK (1982) Noninvasive recording of the Mauthner neurone action potential in larval zebrafish. J Exp Biol 101: 83–92. 7166698 10.1242/jeb.101.1.83

[31] Goldberg JM, Brown PB (1969) Response of binaural neurons of dog superior olivary complex to dichotic tonal stimuli: some physiological mechanisms of sound localization. J Neurophysiol 32: 613–636. 5810617 10.1152/jn.1969.32.4.613

[32] Heil P (2004) First-spike latency of auditory neurons revisited. Curr Opin Neurobiol 14: 461–467 10.1016/j.conb.2004.07.002 15321067

[33] Furukawa S, Middlebrooks JC (2002) Cortical Representation of Auditory Space: Information-Bearing Features of Spike Patterns. J Neurophysiol 87: 1749–1762 10.1152/jn.00491.2001 11929896

[34] Tan X, Wang X, Yang W, Xiao Z (2008) First spike latency and spike count as functions of tone amplitude and frequency in the inferior colliculus of mice. Hear Res 235: 90–104 10.1016/j.heares.2007.10.002 18037595

[35] Eggermont JJ (1998) Azimuth Coding in Primary Auditory Cortex of the Cat. II. Relative Latency and Interspike Interval Representation. J Neurophysiol 80: 2151–2161. 9772268 10.1152/jn.1998.80.4.2151

[36] Heil P (1997) Auditory Cortical Onset Responses Revisited. I. First-Spike Timing. J Neurophysiol 77: 2616–2641. 9163380 10.1152/jn.1997.77.5.2616

[37] VanRullen R, Guyonneau R, Thorpe SJ (2005) Spike times make sense. Trends Neurosci 28: 1–4 10.1016/j.tins.2004.10.010 15626490

[38] Fenno L, Yizhar O, Deisseroth K (2011) The Development and Application of Optogenetics. Annu Rev Neurosci 34: 389–412 10.1146/annurev-neuro-061010-113817 21692661 PMC6699620

[39] Karavitaki KD, Corey DP (2010) Sliding adhesion confers coherent motion to hair cell stereocilia and parallel gating to transduction channels. J Neurosci 30: 9051–9063 10.1523/JNEUROSCI.4864-09.2010 20610739 PMC2932470

[40] Kozlov AS, Risler T, Hudspeth AJ (2007) Coherent motion of stereocilia assures the concerted gating of hair-cell transduction channels. Nat Neurosci 10: 87–92 10.1038/nn1818 17173047 PMC2174432

[41] Trapani JG, Obholzer N, Mo W, Brockerhoff SE, Nicolson T (2009) Synaptojanin1 is required for temporal fidelity of synaptic transmission in hair cells. PLoS Genet 5: e1000480 10.1371/journal.pgen.1000480 19424431 PMC2673039

[42] Farris HE, LeBlanc CL, Goswami J, Ricci AJ (2004) Probing the pore of the auditory hair cell mechanotransducer channel in turtle. J Physiol 558: 769–792 10.1113/jphysiol.2004.061267 15181168 PMC1665030

[43] Jaramillo F, Hudspeth AJ (1991) Localization of the hair cell's transduction channels at the hair bundle's top by iontophoretic application of a channel blocker. Neuron 7: 409–420. 1716929 10.1016/0896-6273(91)90293-9

[44] Gunaydin LA, Yizhar O, Berndt A, Sohal VS, Deisseroth K, et al (2010) Ultrafast optogenetic control. Nat Neurosci 13: 387–392 10.1038/nn.2495 20081849

[45] Eatock RA, Corey DP, Hudspeth AJ (1987) Adaptation of mechanoelectrical transduction in hair cells of the bullfrog's sacculus. J Neurosci 7: 2821–2836. 3498016 10.1523/JNEUROSCI.07-09-02821.1987 PMC6569155

[46] Smedemark-Margulies N, Trapani JG (2013) Tools, methods, and applications for optophysiology in neuroscience. Front Mol Neurosci 6: 18 10.3389/fnmol.2013.00018 23882179 PMC3713398

[47] Faber DS, Korn H (1975) Inputs from the posterior lateral line nerves upon the goldfish Mauthner cells. II. Evidence that the inhibitory components are mediated by interneurons of the recurrent collateral network. Brain Res 96: 349–356. 1175018 10.1016/0006-8993(75)90746-5

[48] Korn H, Faber DS, Mariani J (1974) [Existence of projections of posterior nerves from the lateral line on the Mauthner cell; their antagonistic effect on the activation of this neuron by vestibular afferences]. Comptes Rendus Hebd Séances Académie Sci Sér Sci Nat 279: 413–416. 4373185

[49] Korn H, Faber DS (2005) The Mauthner Cell Half a Century Later: A Neurobiological Model for Decision-Making? Neuron 47: 13–28 10.1016/j.neuron.2005.05.019 15996545

[50] Liao JC, Haehnel M (2012) Physiology of afferent neurons in larval zebrafish provides a functional framework for lateral line somatotopy. J Neurophysiol 107: 2615–2623 10.1152/jn.01108.2011 22338025 PMC3362281

